# Paternally Inherited *P*-Element Copy Number Affects the Magnitude of Hybrid Dysgenesis in *Drosophila simulans* and *D. melanogaster*

**DOI:** 10.1093/gbe/evaa084

**Published:** 2020-04-27

**Authors:** Antonio Serrato-Capuchina, Jeremy Wang, Eric Earley, David Peede, Kristin Isbell, Daniel R Matute

**Affiliations:** e1 Biology Department, University of North Carolina, Chapel Hill; e2 Genetics Department, University of North Carolina, Chapel Hill; e3 Genomics in Public Health and Medicine RTI International, Research Triangle Park, North Carolina

**Keywords:** *P*-elements, *Drosophila*, hybrid dysgenesis

## Abstract

Transposable elements (TEs) are repetitive regions of DNA that are able to self-replicate and reinsert themselves throughout host genomes. Since the discovery of TEs, a prevalent question has been whether increasing TE copy number has an effect on the fitness of their hosts. *P*-elements (PEs) in *Drosophila* are a well-studied TE that has strong phenotypic effects. When a female without PEs (M) is crossed to a male with them (P), the resulting females are often sterile, a phenomenon called hybrid dysgenesis (HD). Here, we used short- and long-read sequencing to infer the number of PEs in the genomes of dozens of isofemale lines from two *Drosophila* species and measured whether the magnitude of HD was correlated with the number of PEs in the paternal genome. Consistent with previous reports, we find evidence for a positive correlation between the paternal PE copy number and the magnitude of HD in progeny from ♀M × ♂ P crosses for both species. Other crosses are not affected by the number of PE copies. We also find that the correlation between the strength of HD and PE copy number differs between species, which suggests that there are genetic differences that might make some genomes more resilient to the potentially deleterious effects of TEs. Our results suggest that PE copy number interacts with other factors in the genome and the environment to cause HD and that the importance of these interactions is species specific.

## Introduction

Transposable elements (TEs) are repetitive elements that are nearly ubiquitous across Eukaryote genomes. TEs can account for a large portion of the genome in both metazoans and plants, making up 69% of the DNA in humans and 85% in maize (Schnable et al. 2009; de Koning et al. 2011), with TE content varying between species and even populations (Anxolabehere et al. 1985; Ronsseray et al. 1989; Stapley et al. 2015; Vicient and Casacuberta 2017). Large genome sizes across the tree of life have been associated with the proliferation of TEs (Kidwell 2002; Hawkins et al. 2006; Chénais et al. 2012). TEs can decrease the fitness of hosts by up to 5% depending on their identity and copy number (Eanes et al. 1988; Pasyukova et al. 2004).

One of the best studied cases of the phenotypic effects of TEs across Eukaryotic systems is *P*-elements (PEs) in *Drosophila* (Kelleher 2016). PEs have rapidly spread worldwide throughout populations of the genetic model system, *Drosophila melanogaster*. The first report of PE carrying *D. melanogaster* individuals was in 1977; PE infection frequencies among populations increased rapidly, and all isolates collected since 1974 carry PEs (Kidwell et al. 1977; Engels and Preston 1980). PEs in *D. melanogaster* lead to F1 sterility when the germline of the female does not carry the repressors that prevents transcription of the transposase gene (Bregliano and Kidwell 1983; Lee et al. 1998; Michalak 2009; Tasnim and Kelleher 2018). When a female who lacks PEs (M) crosses with a male PE carrier (P), the resulting F1s (both females and males) are sterile and show elevated rates of chromosomal breakage and mutation, a suite of traits referred to collectively as hybrid dysgenesis (HD) (Kidwell et al. 1977; Bregliano et al. 1980; Bregliano and Kidwell 1983; Kelleher 2016). If P females mate with P males, the F1s are fertile and show no signs of HD. In this case, the deleterious effects of PEs are silenced through the genetic interaction between PIWI proteins and a maternally inherited and germline-specific subclass of small noncoding RNAs, Piwi-interacting RNAs (piRNAs; Slotkin and Martienssen 2007; Obbard et al. 2009; Siomi et al. 2011; Hadjiargyrou and Delihas 2013; Saito 2013; Weick and Miska 2014).

Recently, PEs were also found in populations of *Drosophila simulans*, another cosmopolitan human commensal of the *melanogaster* species subgroup (Kofler, Hill, et al. 2015). PEs spread into large portions of the *D. simulans*’ geographic range within 15 years (Hill et al. 2016). The phenotypic effects of PEs in *D. simulans*, in the form of sterility of F1s, are similar to those in *D. melanogaster* either in naturally infected populations (Hill et al. 2016) or laboratory produced infections (Daniels et al. 1989; Montchamp-Moreau 1990). Because the hybrid progeny between *D. melanogaster* and *D. simulans* is usually sterile (Sturtevant 1920; Ranz et al. 2004; Turissini et al. 2018; but see Davis et al. 1996; Barbash and Ashburner 2003), there is no obvious genetic bridge via vertical transmission through which PEs could have entered *D. simulans* from *D. melanogaster*. The gene genealogies of PEs found in multiple distant groups of *Drosophila* suggest that the proliferation of PEs has occurred though horizontal gene transfer (Daniels et al. 1990; Houck et al. 1991; de Frutos et al. 1992; Kofler 2019). A natural question is whether the genomes of related species which have been colonized by PEs, in parallel, show conserved patterns in their transmission and intensity of phenotypic effects (Watson and Demuth 2013; Marco et al. 2018; Serrato-Capuchina and Matute 2018).

Despite the large body of work that has addressed the biology of PEs, the study of PE copy variation in individual genomes is an ongoing development. Earlier studies used a variety of techniques (e.g., southern blotting [Brookfield et al. 1984; Black et al. 1987; Good et al. 1989; Itoh and Boussy 2002; Ruiz and Carareto 2003], in situ hybridization to polytene chromosomes [Biémont et al. 1990; Maside et al. 2001], or polymerase chain reaction (PCR) [Onder and Kasap 2014; Ignatenko et al. 2015]) to estimate the number of PE copies in a genome. More recently, genome sequencing has also been used to count the number of PE copies in a genome. The main advantage of whole genome sequencing is that one can infer not only how many copies are present but also to verify where they are inserted. Recent work has compared different computational methods and found that the estimated copy number is highly correlated across different bioinformatics approaches. Several studies have counted the number of PE copies per genome to a high degree of confidence (e.g., Lee and Langley 2010; Bergman et al. 2017; Srivastav and Kelleher 2017).

One of the outstanding questions in our understanding of PEs is to what extent PE copy number (PE_CN_) affects the magnitude of HD similarly across species. Early HD studies showed a positive correlation between PE_CN_ in the parental genome and the severity of HD in crosses between M mothers and P fathers (Bingham et al. 1982; Kidwell 1985; Engels et al. 1987). Engels et al. (1987) modified PEs to be expressed in somatic tissues and suggested that PE_CN_ increased the chances of pupal lethality. The correlation might not be exclusive to PEs. Vieira et al. (1998) found a positive relationship between the number of three TEs (*Penelope*, *Helena*, and *Paris*) and the percentage of atrophied gonads in *Drosophila virilis*. The limitation of these seminal studies was inferring the number of PE copy numbers in the genome. More recently, two efforts have scored the number of PEs using short-read Illumina sequences and have studied the relationship between PE copy number and the severity of HD. In *D. melanogaster*, there seems to be a positive, but weak, relationship between PE number and the proportion of dysgenic female progeny from crosses between ♀M females and ♂P males (♀M/P♂; Eanes et al. 1988; Srivastav and Kelleher 2017). In *D. simulans*, Hill et al. (2016) also reported a correlation between the strength of dysgenic ♀M/P♂ female progeny. However, at least one experimental study (Srivastav et al. 2019) and a meta-analysis of previous studies (Bergman et al. 2017) have argued the correlation is not biologically real, and the magnitude of variation in gonadal dysgenesis across strains of *D. melanogaster* cannot be explained by the number of PEs in the paternal genome . To assess whether the effects of PE (or any other TE) copy number on HD are comparable in different species, systematic measurements of the effect of PEs in HD for the two species in common conditions are sorely needed.

In this report, we estimate the number of PE copies in multiple *D. simulans* and *D. melanogaster* lines. We confirmed the presence of full PEs using PCR and inferred the number of PEs per genome in isofemale lines collected in the island of Bioko using paired-end read data. The results indicate that the number of PEs per genome in our sample ranges from 5 to 25 in each species, approximately. We then measured the magnitude of HD in crosses between P and M lines in *D. simulans*, by scoring for three traits associated with female HD syndromes. We found that HD in *D. simulans* is not restricted to just atrophied gonads in F1 ♀M/P♂ individuals but instead can manifest itself as a continuum in the form of a reduced number of ovaries (consistent with previous reports [Hill et al. 2016]) and reduced ovariole number in nonsterile females. We also find a weakly accelerated reproductive senescence in F1 ♀M/P♂ females. Finally, we tested whether the number of PEs in the paternal genome was correlated with the severity of HD in *D. simulans* and *D. melanogaster*. We found that in both species, the number of PEs in the genome is indeed correlated with the magnitude of the multiple phenotypes associated with the syndrome of HD, but the strength of the correlation differs between phenotypes and species. Our findings show that this correlation is heavily affected by temperature as well. These results add to the body of work that indicates that the magnitude of HD is a complex phenomenon that depends not only on the PE status but also on the species identity, the PE copy number (Bingham et al. 1982; Hill et al. 2016; Srivastav and Kelleher 2017; this report), the maternal genotype (Kelleher et al. 2018; Tasnim and Kelleher 2018), temperature (Kidwell et al. 1977; Bregliano and Kidwell 1983; this report), and the multiple interactions of these factors.

## Materials and Methods

### Stocks and Fly Husbandry

#### Drosophila simulans

We collected 37 *D. simulans* isofemale lines (i.e., lineages derived from the progeny of a single female) across North America and Africa. Supplementary table S1, Supplementary Material online, shows the collection details of each line. The founding females of the isofemale lines were collected using yeasted banana traps (Turissini and Matute 2017). These lines were later inferred to be polymorphic in their PE content (See immediately below). Because *D. melanogaster* and *D. simulans* females look alike in field collections, we started isofemale lines and were only able to distinguish between these two species after they produced male progeny, which differs in their genitalia. We also used two M lines: NC105 (donated by J.A. Ayroles) and MD199 (donated by J.R. David). All lines have been maintained in cornmeal food bottles since their inception.

#### Drosophila melanogaster

We collected *D. melanogaster* lines from Zambia in 2015. We obtained fourteen females that were then maintained as isofemale lines. As expected, all these lines were infected with PEs (see below). All collections, isofemale line establishment, and fly rearing and maintenance were done as described above for *D. simulans*. Additionally, we used seven P lines from the *Drosophila* Genetic Reference Panel and an M subline, Canton-S (scored with PCR, see below). The names and collection details of each line appear in supplementary table S1, Supplementary Material online.

#### Stock Maintenance and Virgin Collection

We kept isofemale lines in 100-ml bottles and collected virgins as previously described. Some inbreeding is expected in these lines (Hoffmann and Parsons 1988; David et al. 2005) but they were not subjected to brother–sister matings. We cleared adults every 8 h, as soon as we saw black pupae in the bottles. We separated females and males and housed them in sex-specific vials in groups of ∼20 individuals. On day 4, we inspected the vials to make sure they had not produced progeny. If a vial contained larvae, we discarded the vial. Then, we proceeded to mix females and males from different genotypes. We checked the vials every 2–3 days looking for larvae. As soon as we saw L3 larvae, we added propionic acid and a pupation substrate (Kimwipes Delicate Task; Kimberly Clark, Roswell, GA) to the vial. We inspected the vials on a regular basis until we saw pupae and collected the F1s from the crosses in the same way we collected pure species virgins. These F1s were aged to 4 days and kept as virgins for female reproductive tract dissections.

### PE Detection

We used three different methods to detect PEs in the genomes of the *D. simulans* and *D. melanogaster* lines described above: Illumina short reads, PCR, and Oxford Nanopore Technology single-molecule sequencing. We describe the goals and procedures for each of the three approaches.

- *Short reads (Illumina)*: We obtained paired-end Illumina reads for each isofemale line included in this study from the two species (*D. simulans*: *N* = 37*, D. melanogaster*: *N* = 21). For *D. simulans*, we obtained sequence data for each isofemale line mentioned in the study. First, we obtained genomic DNA using the QiagenDNeasy 96 Tissue Kit following the protocol and recommendations from the manufacturer. Next, we outsourced library construction and sequencing to the High Throughput Sequencing Facility at the University of North Carolina, Chapel Hill. The libraries were built using the Nextera protocol as specified by the manufacturer. We obtained read quality information using HiSeq Control Software 2.0.5 and RTA 1.17.20.0 (real time analysis). CASAVA-1.8.2 (Illumina, Hayward, CA) generated and reported run statistics of each of the final FASTQ files. Resulting reads ranged from 100 to 150 bp, and the target average coverage for each line was 30×. The sequencing coverage for the studied lines ranged between 24.68× and 94.27×; Supplementary table S1, Supplementary Material online, shows the coverage for all studied lines. We repeated the same procedure for *D. melanogaster* and used public data and sequencing data for a total of 21 lines. The procedure to obtain de novo sequencing for *D. melanogaster* was similar to the description above. We used seven previously published genomes (Sequence Read Archive, numbers in supplementary table S1, Supplementary Material online) and generated reads for fourteen more lines. The coverage per line range between 22.73× and 258.60× with a median of was 42×; Coverages for each genome are reported in supplementary table S1, Supplementary Material online.
- *PCR assays*: We assessed whether individuals from the lines included in this study had any of the four exons that constitute a full PE. Because PEs require all four exons to be functional, our goal was to type all the individuals for each exon individually using PCR. We extracted genomic DNA from one female of each isoline following the 96-well Gentra Puregene extraction kit protocol. To individually amplify each of the four exons that make up the full PE, we used primers described in Hill et al. (2016) with both positive and negative controls during each run. We did all PCRs using NEB reagents in a 10-µl reaction (1 µl 10× buffer, 1 µl 10 mM MgCl_2_, 0.5 µl 10 mM dNTPs, 0.3 µl 10 mM F + R primers, 1 µl DNA, 0.05 µl Taq Polymerase, and 5.85 µl H_2_O) with a thermocycling cycle of 92**°** denaturing, 59**°** annealing, and 72**°** extension for 35 cycles in an Applied Biosystems 2720 Thermal Cycler. To score presence/absence of each exon, we ran 5 µl of the PCR product in a 2% (APExBIO) agarose gel for 60 min at 120 V and visualized the results using ethidium bromide staining. Sanger sequencing (Eurofins) was used to verify PE presence in isolines that amplified for each primer to ensure the presence of the full continuous element.
- *Single-molecule sequencing*: Finally, we performed Oxford Nanopore sequencing from a representative subset of lines listed in supplementary table S2, Supplementary Material online. DNA was extracted from 20 whole male flies of each line using a modified phenol:chloroform protocol (Sambrook and Russell 2006). We prepared libraries using the Ligation Sequencing Kit 1D with native barcoding (SQK-LSK109 and EXP-NBD104, Oxford Nanopore) according to the manufacturer’s protocol. Libraries were sequenced on six R9.4 flow cells with an average of 1,526 available pores and run for 48 h, or until no pores were available, on a GridION running MinKNOW v3.1.8. Reads were basecalled using Guppy (ONT) with dna_r9.4.1_450bps_flipflop model. For each individual line, we mapped reads in a pairwise fashion using Minimap2 v2.15-r905 (Li 2018) and assembled them using Miniasm v0.3-r179 (Li 2016). We then corrected and generated consensus assemblies with four iterations of Racon v1.3.2 (Vaser et al. 2017) followed by Medaka v0.6.2 (https://github.com/nanoporetech/medaka).

### PE Copy Number (PE_CN_) Estimation

The procedure to determine whether lines were infected with PEs was identical for the two species. We aligned the paired-end Illumina reads to the canonical *D. melanogaster* PE (https://flybase.org/reports/FBte0000037.html) using minimap2 in short-read mode (“-cx sr”) and to the *D. simulans* reference sequence r2.02 (ftp://ftp.flybase.net/genomes/Drosophila_simulans/dsim_r2.02_FB2017_04/fasta/dsim-all-chromosome-r2.02.fasta.gz). The number of copies per genome was calculated by dividing mean read coverage of the PE sequence by the mean read coverage per genome. This approach allowed us to infer how many PE copies each sampled isofemale line harbored. All lines that showed fewer than 0.5 copies of the PE (i.e., a single heterozygote copy) were considered PE-negative. Additionally, we inferred the PE insertion sites by detecting “split” reads that overlap a terminal portion of the PE and a portion of the host genome. We aligned Nanopore long reads to the PE and reference genome as described above (except using minimap2 parameter “-cx map-ont” to sensitively align erroneous long reads). If PEs (either partial or complete) are present in a genome, long reads are expected to completely encompass the PE sequence and include the anchoring genomic sequence on either side (supplementary fig. S1, Supplementary Material online). We used the same approach for all studied lines.

### *Hobo* Detection and Copy Number Estimation

To test if overall TE copy number can affect HD, we included another well-characterized TE, *hobo*, into our analysis (Blackman et al. 1987). *hobo* TE can cause HD in *D. melanogaster*, independent from PEs (Yannopoulos et al. 1987; Stamatis et al. 1989). We scored the number of copies of *hobo* elements in each genome by following an identical approach to the one described to detect PEs using genomic data (immediately above) using short-reads and Nanopore data. Alignments were done using the *D. melanogaster hobo* sequence (http://flybase.org/reports/FBgn0014191.html).

### HD in *D. simulans*

The most pronounced phenotype of PEs in *D. melanogaster* is HD; when M females are crossed to P males, the female progeny often shows atrophied ovaries. Similarly, Hill et al. (2016) found that *D. simulans* F1 females resulting from the same direction of the M females × P males are likely to show the same phenotypic defect, atrophied ovaries. Females resulting from any of the other three directions of the cross (♀P × ♂P, ♀P × ♂M, and ♀M × ♂M) have functional ovaries. In *D. melanogaster*, HD has been extensively studied, and in addition to the number of atrophied ovaries, other defects have been associated with the HD syndrome, namely, reduced number of ovarioles in females with functional ovaries, and male sterility (Kidwell et al. 1977). In *D. simulans*, HD is known to cause a reduced number of ovaries. We expanded these studies by studying the effect of PE presence on *D. simulans* with respect to three aspects of F1 female fecundity: the number of functional ovaries, the mean number of ovarioles per ovary, and the rate of reproductive senescence. (Please note that the rate of F1 embryo hatchability increases with female age [Bucheton 1979; Khurana et al. 2011], but we scored the total number of ovarioles instead.) To reduce the possibility of line effects, we studied the three phenotypes in F1s from four different lines, 2 P (Riaba and Karitana06) and 2M (NC105 and MD199) and carried out all the possible crosses in a diallelic cross at two temperatures (23 and 29 °C). Details of each line of the four lines are listed in supplementary table S1, Supplementary Material online. We describe the procedure to score the three phenotypes as follows.

#### Crosses

Our goal was to assess whether ♀M × ♂P (♀M/P♂) F1s had lower fecundity than other genotypes of F1s. We crossed M and P lines in the four possible combinations: ♀P × ♂P (♀P/P♂), ♀P × ♂M (♀P/M♂), ♀M × ♂M (♀M/M♂), and ♀M × ♂P (♀M/P♂). (For all crosses, the female genotype is shown first.) To make F1s, we collected virgins as described above (section “Stock maintenance and virgin collection”) and mixed virgin males and females. Once we observed pupae, we cleared the vials every 8 h and collected virgin flies. These virgins were then aged to 4 days (unless otherwise specified) and we used them for experimentation.

#### Ovary Number: Counts

We studied the fecundity of 4- to 7-day-old virgin F1 females. First, we scored whether F1 individuals had 0, 1, or 2 developed ovaries, with healthy females having two ovaries, at both 23 and 29 °C. After 4–9 days, the peak age of sexual maturity (Wayne and Mackay 1998; Wayne et al. 2006), virgin females were anesthetized with CO_2_ and their gonads were removed with metallic forceps (Wong and Schedl 2006). Gonads from each individual were subsequently fixed on a precleaned glass slide with chilled *Drosophila* Ringer’s solution (Cold Spring Harbor Protocols). We counted the number of nonatrophied ovaries for each individual. Ovaries were considered atrophied if they had no ovarioles. We also counted the number of ovarioles (see below) in each mature ovary using a Leica, S6E stereoscopic microscope. We scored >100 females at 23 °C and >40 females at 29 °C, as higher temperatures are associated with an increase of TE transposition (Kidwell et al. 1977; Bregliano et al. 1980; Schoville et al. 2012). Females with at least one atrophied ovary were considered dysgenic. In total, we did 16 types of crosses and assayed 50 females per cross for a total of 1,600 females (16 combinations × 2 temperatures × 50 females per cross).

#### Ovary Number: Statistical Analyses

We scored whether each F1 female had 0, 1, or 2 ovaries as described above. To quantify the magnitude of heterogeneity among F1 genotypes, we fitted a multinomial regression using the function *multinom* in the library “*nnet*” (Venables and Ripley 2003) where the number of ovaries was the response of the interaction between the genotypes of the two parentals to account for the interplay between the genome of the two parents. The significance of the effects was inferred using a type III ANOVA (function *Anova*, library “*car*”; Fox and Sanford 2011) in R. Because we did experiments at two different temperatures (23 and 29 °C), we fitted two multinomial regressions both of which took the form:

$$\mathrm{Number}\;\mathrm{of}\;\mathrm{ovaries}\;∼\;{\left({\mathrm{genotype}}_{\mathrm{father}}\times {\mathrm{genotype}}_{\mathrm{mother}}\right)}_{ij}+{\mathrm{Error}}_{ij}.$$

To do post hoc comparisons between crosses, we used a Two-Sample Fisher–Pitman Permutation Test (function *oneway_test*, library “*coin*”; Hothorn et al. 2006) and adjusted the critical *P* values for significance to 0.008 to account for multiple comparisons (six comparisons). Finally, we fitted a model to study the triple interaction between parental genotypes and temperature. The model included all observations, only included the triple interaction, and took the form:

$$\mathrm{Number}\;\mathrm{of}\;\mathrm{ovaries}\;∼\;{\left({\mathrm{genotype}}_{\mathrm{father}}\times {\mathrm{genotype}}_{\mathrm{mother}}+{\mathrm{Error}}_{\mathrm{ijk}}\times \;\mathrm{temperature}\right)}_{\mathrm{ijk}}.$$

For all linear models (including the ones that follow), we used a maximum-likelihood model simplification (Crawley 1993, 2007) in which the full model containing all factors and interactions was fitted and then simplified by a series of stepwise deletions, starting with the highest-order interaction and progressing to lower-order interaction terms and then to main effects.

#### Ovariole Number: Statistical Analyses

A second potential phenotype of HD is the reduction in the number of ovarioles per ovary in female F1s that did not show atrophied ovaries. Even with two ovaries, these females can have limited reproductive potential through a reduction of ovarioles within ovaries. We scored whether the genotype of the mother, the genotype of the father, or the interaction between these two terms affected the number of ovarioles. We analyzed the mean number of ovarioles per ovary (i.e., females with two ovaries will have more total ovarioles than females with one ovary) to account for the difference in the number of ovaries. We excluded those females that showed completely atrophied ovarioles from this analysis because they contain zero ovarioles. The analyses were similar to those described for ovary number with the only difference being that we used a Poisson-distributed linear model (function *glm*, library “*stats*”; R-Core-Team 2013) for each temperature instead of a multinomial response.

#### Female Reproductive Senescence: Counts

We tested whether the age of the female affected the number of ovarioles in P and M females. Specifically, we explored whether HD manifested itself as a shorter reproductive period in potentially dysgenic genotypes. In this scenario, ♀M/P♂ F1 females will show a sharper decline in their ovariole number compared with females from other PE-related genotypes. To score females of different ages, we cleared bottles and collected newly eclosed virgins within 8 h of clearing as described above (section “Crosses”) and let them age up to 29 days posteclosion. Female virgins were then dissected every 5 days (six time points: 4, 9, 14, 19, 24, and 29 days), and we counted the ovariole number as they aged. We counted 50 females per line combination at each temperature. In total there were 1,920 observations: 6 time points × 8 line combinations × 20 individuals per line × 2 temperatures.

#### Female Reproductive Senescence: Statistical Analyses

We used an analysis of covariance to assess whether the presence of PEs affected the reproductive capacity of females of different ages by scoring the mean number of ovarioles of the four possible F1 genotypes over the course of 29 days. Similar to the experiments described above, we did these experiments at two temperatures: 23 and 29 °C. The crossing scheme followed the same diallelic design described above. For each line combination, and at each temperature, we collected ∼120 females and split them into six groups. Each group was dissected every 5 days (starting at day 4 post collection). The time points then were 4, 9, 14, 19, 24, and 29 days old. We used the function *glm* in the R library “*stats*” (R-Core-Team 2013). We fitted two models—one that included the interaction between the genotype of the F1s and the age:

$$\mathrm{Model}\;1:\;\mathrm{Mean}\;\mathrm{number}\;\mathrm{of}\;\mathrm{ovarioles}\;∼\;{\mathrm{age}}_{i}+{\mathrm{cross}}_{j}+{\left(\mathrm{age}\times \mathrm{cross}\right)}_{ij}+{\mathrm{Error}}_{ij}$$

and another one without the interaction:

$$\mathrm{Model}\;2:\;\mathrm{Mean}\;\mathrm{number}\;\mathrm{of}\;\mathrm{ovarioles}\;∼\;{\mathrm{age}}_{i}+{\mathrm{cross}}_{j}+{\mathrm{Error}}_{ij}.$$

We used these two models to compare the rate of decline of fertility among F1 genotypes as age progressed by comparing the two models with a likelihood ratio test (LRT; function *lrtest*, library “*lmtest*”; Kuznetsova et al. 2015). Second, we used the regression coefficients from the model that better fit the data to compare the intercept of the linear regressions of females of the four F1 genotypes. This test assessed whether genotypes had inherent differences in the number of ovarioles. To infer the significance of the main effects, we used a type III ANOVA (function *Anova*, library “*car*”; Fox and Sanford 2011) in R.

### The Effect of PE_CN_ on the Strength of HD

Our second experiment was tailored to detect whether PE_CN_ affected the magnitude of HD in both *D. simulans* and *D. melanogaster*. To study the effect of PE_CN_ in HD in *D. simulans*, we crossed each of 37 *D. simulans* lines to four different lines (listed in supplementary table S1, Supplementary Material online), two of which were M (NC105 and MD199) and two of which were P (Riaba and Karitana06). We followed a similar approach for *D. melanogaster*. We crossed each of the 21 *D. melanogaster* lines to two different lines, one M (Canton-S) and one P (DGRP385). The details of all the lines are listed in supplementary table S1, Supplementary Material online. The procedure to carry crosses for this set of experiments was similar to that described above to detect effects of PEs within *D. simulans*. Fly husbandry and virgin collection were identical in the two experiments. The expectation was that if PE_CN_ increases HD, then the number of ovaries and mean ovarioles per ovary per female should decrease as the number of paternal PE copies increases, but only in crosses that involve M females. In crosses with P females, increasing the number of PE copies in the paternal genome should have no effect on phenotypes affected by HD. We scored ovary number and mean ovariole number per ovary per female, as described above (section HD phenotypes in *D. simulans*) for 50 females per line combination.

#### Ovary Number versus PE_CN_: Statistical Analyses

We scored whether each F1 female had 0, 1, or 2 ovaries as described above. For each of the temperatures and mother genotype combinations, we fitted a linear model for a total of four linear models per species. To quantify the magnitude of heterogeneity among F1 genotypes, we fitted a multinomial regression using the function *multinom* in the library “*nnet*” (Venables and Ripley 2003) where the number of ovaries was the response of the multinomial assay, and the number of PEs and *hobo* elements in the paternal genome were continuous effects. We also included an interaction between these two effects. The linear models took the form:

$$\mathrm{Ovary}\;\mathrm{number}\;∼{\;\mathrm{PE}\;\mathrm{copy}\;\mathrm{number}}_{i}+{\mathrm{hobo}\;\mathrm{copy}\;\mathrm{number}}_{j}+{\left(\mathrm{PE}\;\mathrm{copy}\;\mathrm{number}\times \mathrm{hobo}\;\mathrm{copy}\;\mathrm{number}\right)}_{ij}+{\mathrm{Error}}_{ij}.$$

For each of the eight linear models (two species × two temperatures × female genotypes), the significance of the effects was inferred using a type III ANOVA (function *Anova*, library “*car*”; Fox and Sanford 2011) in R.

#### Ovariole Number versus PE_CN_: Statistical Analyses

A second potential phenotype of HD is the reduction in the number of ovarioles per ovary in female F1s that did not show atrophied ovaries (Khurana et al. 2011). Even with two ovaries, these females can have limited reproductive potential through a lack of ovarioles (Lobell et al. 2017). We quantified whether the genotype of the mother, the genotype of the father, or the interaction between these two terms affected the number of ovarioles. We analyzed the mean number of ovarioles per ovary (i.e., females with two ovaries will have more total ovarioles than females with one ovary) to account for the difference in the number of ovaries. Noninteger means were approximated to the nearest integer. We excluded those females that showed completely atrophied ovaries from this analysis because they contain zero ovarioles. We used a Poisson-distributed linear model (function *glm*, library “*stats*”; R-Core-Team 2013) in which the mean number of ovarioles per female was the response. We fitted four linear models: four linear models (two species × two temperatures). The linear models took the form:

$$\mathrm{Mean}\;\mathrm{number}\;\mathrm{of}\;\mathrm{ovarioles}\;∼{\;\mathrm{PE}}_{\mathrm{CN}-i}+{\mathrm{genotype}}_{\mathrm{mother}-j}+{\mathrm{hobo}\;\mathrm{copy}\;\mathrm{number}}_{k}+{\left({\mathrm{PE}}_{\mathrm{CN}}\times \;{\mathrm{genotype}}_{\mathrm{mother}}\right)}_{ij}+{\left({\mathrm{PE}}_{\mathrm{CN}}\times \;\mathrm{hobo}\;\mathrm{copy}\;\mathrm{number}\right)}_{ik}+{\left({\mathrm{genotype}}_{\mathrm{mother}}\times \;\mathrm{hobo}\;\mathrm{copy}\;\mathrm{number}\right)}_{jk}+{\left({\mathrm{PE}}_{\mathrm{CN}}\times {\mathrm{genotype}}_{\mathrm{mother}}\times \;\mathrm{hobo}\;\mathrm{copy}\;\mathrm{number}\right)}_{\mathrm{ijk}}+{\mathrm{Error}}_{\mathrm{ijk}}.$$

### Interaction between PE_CN_ and Temperature

Our experiments (see Results) and others (Kidwell et al. 1977; Bingham et al. 1982; Kidwell 1983) indicate that HD is more pronounced at higher temperatures. Our results also suggest that increasing PE_CN_ leads to more severe HD. We explored the interaction between these two factors on the magnitude of HD by scoring HD using the number of ovaries per female and the mean number of ovarioles per ovary per female. As described for previous analyses, atrophied ovaries were considered missing data. We measured this set of traits for a reduced number of lines at a range of temperatures experienced by *Drosophila* in nature. For *D. simulans*, we used ten paternal lines which range from 0 (i.e., M type) to 11.91 PE copies, and four maternal lines: two of which were P type and two were M type. We used a similar experimental design for *D. melanogaster*, but for this species, we only used one M line and one P line. The range of PE copies for this experiment was 0 (i.e., M type) to 20.39 PE copies. We scored HD at seven different temperatures: 17, 19, 21, 23, 25, 27, and 29 °C. We scored 50 females per cross. In total, we scored 5,600 females for *D. simulans* (20 replicates × 40 line combinations × 7 temperatures) and 2,800 for *D. melanogaster* (20 replicates × 20 line combinations × 7 temperatures). To study the interplay between PE_CN_ and temperature, we fitted mixed linear models for each of the two HD metrics. We used parallel analyses for M and P females. In total, we fitted six linear models, which we describe as follows.

For the number of ovaries per female, we used a multinomial logistic model using the R function *multinom* (library *nnet*) with identical continuous factors as the model described above. For the mean number of ovarioles per female, we approximated decimal numbers to the upper nearest integer and fitted a linear regression with Poisson-distributed errors using the function *glm* (library *stats*; R-Core-Team 2013). The two types of linear models followed similar forms:

$$\mathrm{Ovary}\;\mathrm{number}\;∼{\left({\mathrm{genotype}}_{\mathrm{mother}}\times {\mathrm{PE}}_{\mathrm{CN}}\times \mathrm{temperature}\right)}_{\mathrm{ijk}}\;+{\mathrm{Error}}_{ij}$$

and

$$\mathrm{Mean}\;\mathrm{number}\;\mathrm{of}\;\mathrm{ovarioles}\;∼\;{\left({\mathrm{genotype}}_{\mathrm{mother}}+{\mathrm{Error}}_{\mathrm{ijk}}\times {\mathrm{PE}}_{\mathrm{CN}}\times \;\mathrm{temperature}\right)}_{\mathrm{ijk}}.$$

Finally, we compared the strength of the interaction between the mother genotype, the number of PE copies, and temperature in *D. simulans* and *D. melanogaster*. We calculated the proportion of explained variance (*R*^2^) for each generalized linear model described immediately above with the *Dsquared* function (library “*modEvA*”). To compare the *R*^2^ values of the two models, we generated bootstrap distributions of the *R*^2^ values by subsampling the data sets using the functions *sample* and *replicate*. We compared the two bootstrapped distributions using a permutation-based Wilcoxon test implemented in the function *compare.2.vectors* (library “*afex*”).

## Results

### PE and *Hobo* Copy Number and Their Insertion Sites Vary among *D. simulans* Lines

First, we characterized each of the 37 *D. simulans* lines according to whether they were P or M type (i.e., whether they carry PEs or not, respectively). Both PCR and mapping of Illumina reads suggest that, consistent with the rapid expansion of PEs in *D. simulans*, the majority of lines were P type. Notably, none of the lines collected in Africa after 2013 were M, which is consistent with a previous report in *D. simulans* (Hill et al. 2016) and mirrors the rapid spread of PEs in *D. melanogaster* (Anxolabehere et al. 1985; Eanes et al. 1992; Vetorazzi et al. 1999; Kapun et al. 2018). All of the studied lines were positive for *hobo*. We explored three aspects of the PE and *hobo* invasion: 1) mean copy number per genome, 2) proportion of complete copies, and 3) insertion sites.

#### *Mean PE and* Hobo *Copy Number per Genome*

Using paired-end Illumina reads, we found that the inferred number of PEs varied across lines within species. The estimated PE_CN_ in *D. simulans* ranged from 0 to 11.98 PE copies (mean PE_CN-__*sim*_ = 6.589, SD = 1.772). Similar analyses suggest that the estimated PE_CN_ in *D. melanogaster* ranged from 0 to 20.39 (mean PE_CN-__*mel*_ = 7.893, SD = 3.718). We found no differences in PE_CN_ between the two species (Wilcoxon rank sum test with continuity correction: *W* = 566.5, *P* = 0.064).

**Fig. 1.**
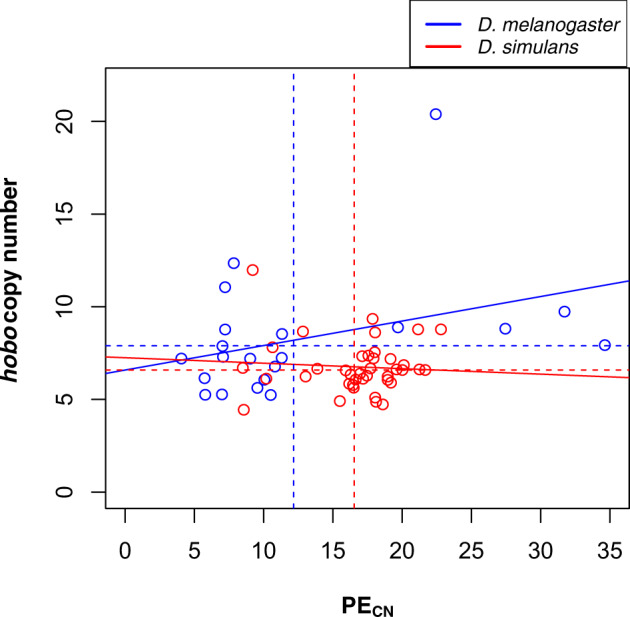
—PE_CN_ and *hobo* copy number are not correlated in *D. simulans* or *D. melanogaster.* The solid lines show the relationship for each of the two species, they were not significant in either species (*D. melanogaster*: *t* = 1.641, df = 19, *P* = 0.117; *D. simulans*: *t* = −0.426, df = 37, *P* = 0.673). Dashed lines show the mean number of PEs or *hobo* elements. Blue: *D. melanogaster*; red: *D. simulans*.

*Hobo* elements were present in all *D. simulans* and all *D. melanogaster* lines, except one (Canton-S; evaluated by PCR). The estimated number of *hobo* in *D. simulans* ranged from 8.50 to 21.15 (mean number of copies_*sim*_ = 16.639, SD = 3.390). Similar analyses suggest that the estimated number of *hobo* in *D. melanogaster* ranged from 0 to 34.63 (mean number of copies_*mel*_ = 12.737, SD = 8.938)*.* We found that the *hobo* copy number differs between the two species (Wilcoxon rank sum test with continuity correction: *W* = 602, *P* = 0.003). The copy number of PEs and *hobo* are not correlated in any of the two species (fig. 1).

**Fig. 2.**
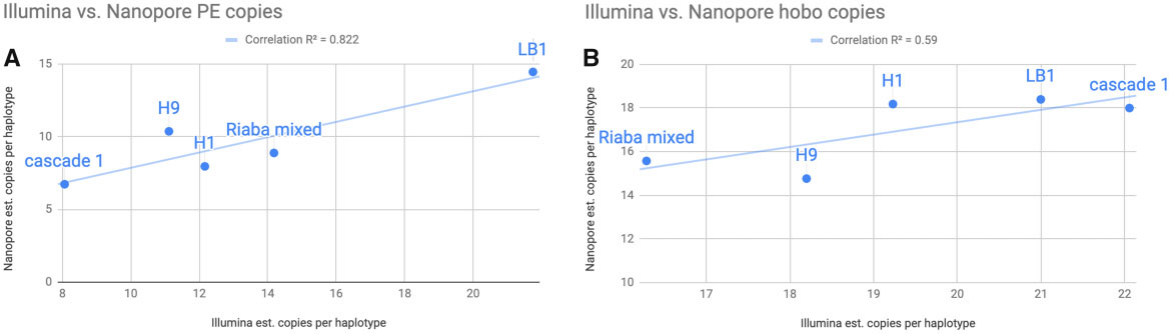
—PE and *hobo* copy number predictions from short-read data and Nanopore counts are correlated. We sequenced individuals from five isofemale lines using short-read sequencing and long-read sequencing and scored the PE and *hobo* copy number. (*A*) Correlation between PE copy number inferred with the two approaches. The correlation between the two approaches was highly significant (Pearson’s *R*^2^ =0.82; *P* = 0.015). (*B*) Correlation between *hobo* copy number inferred with the two approaches. The correlation between the two approaches was not significant (Pearson’s *R*^2^ = 0.59; *P* = 0.13), in part due to lower total variation in *hobo* count across lines (∼16–22) compared with PE (∼8–22).

We selected a group of six *D. simulans* lines, sequenced them with single-molecule sequencing (Nanopore), and estimated the number of PE copies present in their genomes. The copy number is not identical to the number inferred by Illumina reads, but the two numbers are correlated (fig. 2). The differences can be caused by either methodological (sequencing) differences or by true biological differences as flies within a line were sequenced at different generations and to different sequencing depth. Further, the number of PE copies estimated from the Illumina sequencing is based on the expected size of the inserted element and does not take into account the full variability in partial copies. In spite of the differences, these results suggest that using paired-ended short reads is a good proxy of the relative number of PEs (and to a lesser extent the relative number of *hobo*) in a given line compared with the rest of the population.

#### Proportion of Complete Copies

**Fig. 3.**
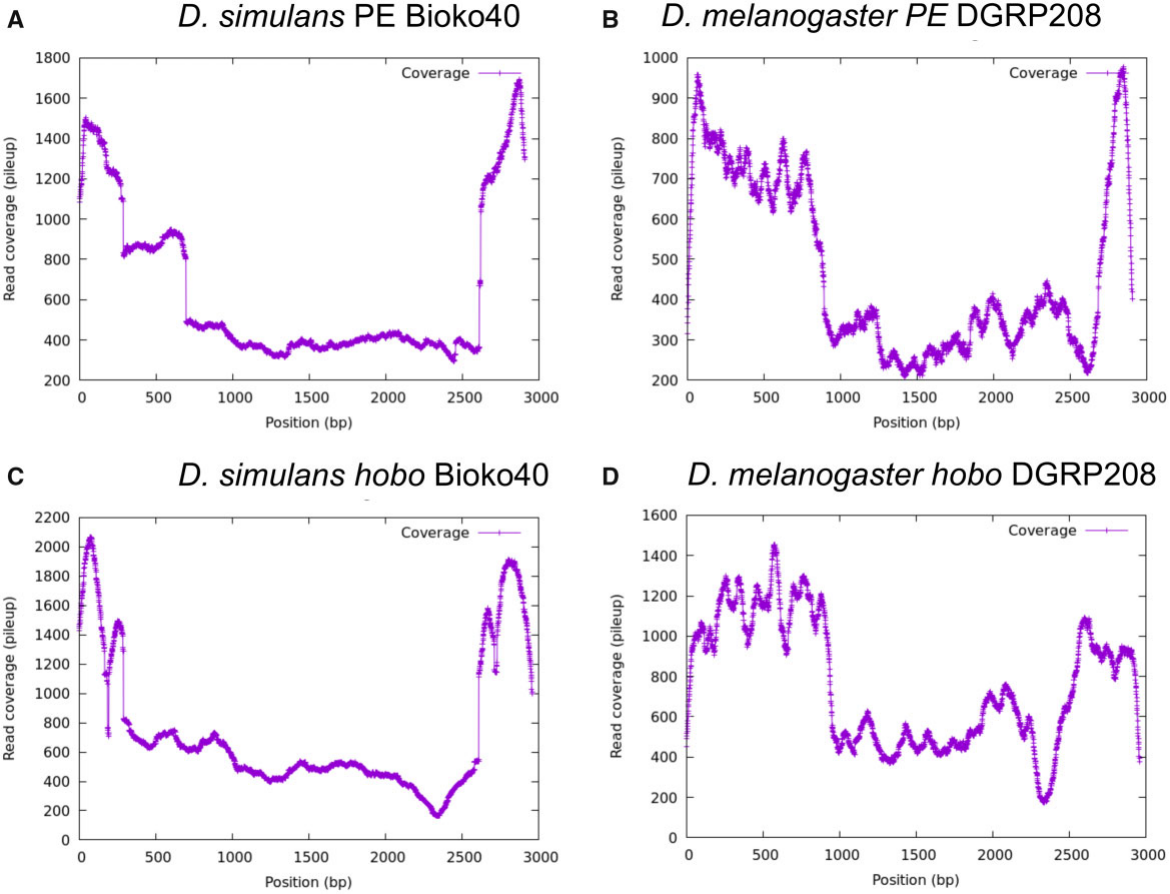
—Incomplete PE and *hobo* elements are common in *D. simulans* and *D. melanogaster* genomes. The *y* axis shows the mean coverage per site along the two elements. (*A*) PE in *D. simulans*. (*B*) PE in *D. melanogaster*. (*C*) *hobo* in *D. simulans*. (*D*) *hobo* in *D. melanogaster*.

Next, we studied what proportion of PEs were complete by counting the number of reads that supported each site across the PE sequence using the short-read and the Nanopore data sets. If all (or most) of the copies are intact, then one would observe a uniform coverage distribution along the PE sequence. If, on the other hand, some segments are absent in some of the PE copies, then the coverage in those segments will be lower. The results for one of the lines (*D. simulans* Bioko40) are shown in figure 3*A*; we find that the results are consistent across lines (supplementary fig. S2, Supplementary Material online). PE exons 0 and 3 are more common (4-fold in average) than exons 1 and 2. This is similar to observations in *D. melanogaster*, where these two center exons are deleted more frequently than the terminal pieces of the PE (fig. 3*B* and supplementary fig. S3, Supplementary Material online). Notably, exon 3 is also more prevalent in *Drosophila willistoni*, another species that harbors PEs (supplementary fig. S4, Supplementary Material online). (We found no evidence for *hobo* in this species.) We examined whether the increased relative abundance of exons 0 and 3 in *D. simulans* was caused by the presence of truncated PEs. Nanopore sequences confirmed that incomplete copies containing only exons 0 and 3 are three times more common than complete copies.

We observed a similar pattern for *hobo*. Most copies were incomplete, and the most common segments were the terminal ends of the sequence in both, *D. simulans* (fig. 3*C* and supplementary fig. S5, Supplementary Material online) and *D. melanogaster* (fig. 3*D* and supplementary fig. S6, Supplementary Material online). Similar to our observations regarding PEs, Nanopore reads suggest that these segments are more common because there are incomplete copies that only include these sequences.

#### Insertions Sites of PEs

On average, each PE insertion site was supported by 9.8 read pairs. We identified 1,311 exact and 118 approximate insertion positions, of which 45 are shared by multiple lines. In total, 1,215 sites were unique to a line, 40 sites were shared by 2 lines, 4 sites were shared by 4 lines, and 1 site was shared in 4 of the 6 unique lines (supplementary fig. S7, Supplementary Material online). Because 20 individuals were pooled to sequence each line, we can infer the average frequency of any single insertion is 1,311 sites/6 lines/40 haplotypes/6.6 mean PE copies = 0.827 haplotype-sites per copy, indicating that, within a margin of error, most insertion sites are unique to a single haplotype within the pool of 40 sequenced chromosomes from each isofemale line.

### HD Caused by PEs in *D. simulans* Is a Multiphenotype Syndrome

Previous reports have found evidence of HD within *D. simulans* in the form of ovary number reduction (Hill et al. 2016). We explored whether F1 *D. simulans* from crosses between M and P lines showed evidence of HD in other phenotypes as well, namely, the mean number of ovarioles per ovary and reproductive senescence at two different temperatures. We report the results of each phenotype as follows.

#### Number of Ovaries

**Fig. 4.**
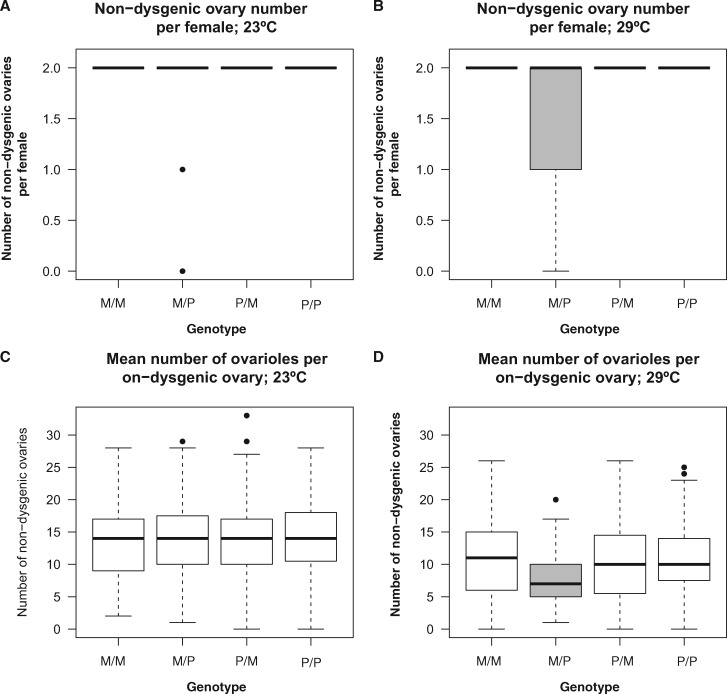
—Hybrid dysgenesis in *D. simulans* takes the form of reduced number of ovaries per females and reduced number of ovarioles per ovary per female at 29 °C but not at 23 °C. (Genotype of the mother is listed first in all cases.) **(***A*) Number of ovaries per female at 23 °C. (*B*) Number of ovaries per female at 29 °C. (*C*) Number of mean ovarioles per ovary per female at 23 °C. (*D*) Number of mean ovarioles per ovary per female at 29 °C. We compared the magnitude of paired crosses using a linear model in which the only effect was the interaction of the genotypes using a linear model that only incorporated the interaction term. As expected by hybrid dysgenesis, ♀M/P♂ progeny has in average fewer ovaries and ovarioles per ovary than the other three types of F1 females at 29 °C (*B* and *D*). We found no significant difference between any of the other pairwise comparisons (table 2). Genotypes with gray boxes show significantly lower means than other genotypes.

We assessed whether the interplay between the mother and father PE-genotypes affected the number of functional ovaries in F1 females. We pooled all lines into genotypic categories to perform a multinomial regression for each of the two assayed temperatures. We find that there is no noticeable effect of the interaction of the parent genotypes in ovary number at 23 °C (LRT: *χ*^2^ = 8.344, df = 8, *P* = 0.401, fig. 4*A*; regression coefficients in supplementary table S3, Supplementary Material online). On the other hand, the interaction between parent genotypes has a strong effect on ovary number at 29 °C (LRT = 215.76, df = 8, *P* < 1 × 10^−10^; regression coefficients in supplementary table S3, Supplementary Material online). We used permutation-based pairwise comparisons to determine whether F1 *D. simulans* from the ♀M × P♂ cross had fewer ovaries than females from any of the other three possible genotypes. We find that, indeed, females from this cross have, on average, fewer ovaries than the rest of the possible crosses (approximative Two-Sample Fisher–Pitman Permutation Test, *Z* = −8.423, *P* < 1 × 10^−10^; fig. 4*B*). All females from the other three crosses had two ovaries and did not differ among themselves (supplementary table S4, Supplementary Material online). This result is in line with a previous report which found that P *D. simulans* males can induce HD when mated to M females (Hill et al. 2016). Finally, we also fitted a linear model that included the interaction between the mother and father genotypes with temperature and found that this interaction was highly significant (LRT: *χ*^2^ = 303.9, df = 8, *P* < 1 × 10^−10^). This result suggests that temperature is an important factor in determining the magnitude of HD in the form of ovary number.

#### Number of Ovarioles

HD can manifest itself not only as the absence of ovaries but also through the development of “rudimentary” ovaries, that is, ovaries with fewer ovarioles (Khurana et al. 2011; Hill et al. 2016). Because F1s in *D. simulans* crosses might lack ovaries depending on the genotype of the parents (e.g., ♀M/P♂), we used the mean number of ovaries per individuals with instances of atrophied ovaries treated as missing data. We found that just as is the case with ovary number, ovarioles are affected by HD but only at 29 °C. At 23 °C, the variation among genotypes was not noticeable (fig. 4*C*), but at 29 °C, we found that the father genotype had a much larger effect size than the mother effect (genotype_mother_: LRT: *χ*^2^ = 0.568, df = 1, *P* = 0.451; PE_CN_, LRT: *χ*^2^ = 89.071, df = 1, *P* < 1 × 10^−10^; fig. 4*D*). The interaction terms between the two effects were also significant (genotype_mother_ × PE_CN_: LR = 58.204, df = 1, *P* = 2.363 × 10^−14^), suggesting that the number of ovarioles depends on the combination of genotypes of the two parents. In a fully factorial linear model (including the genotypes of the parents and temperature) and as observed for ovary number, temperature affected the magnitude of the interaction between parental genotypes (LRT: *χ*^2^ = 614.17, df = 4, *P* < 1 × 10^−10^), a result that is explained by the existence of HD only at 29 °C (fig. 4*D*).

#### Reproductive Senescence

**Fig. 5.**
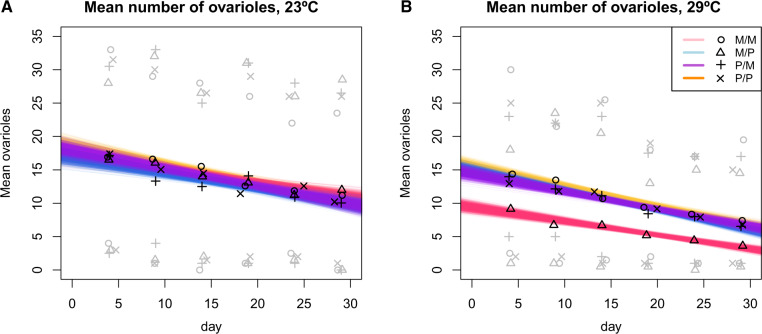
—Number of ovarioles observed in *D. simulans* females as they age at two different temperatures. The four different colors represent a type of cross: blue: ♀M/M♂; purple: /P; yellow: ♀P/M♂; and pink: ♀M/P♂. Black symbols show the mean value for a cross at a given day. Gray symbols show the minimal and maximum number observed for each cross at a given day. To estimate 95% confidence intervals, we bootstrapped the linear regression for each genotype 1,000 times. (*A*) Decline of fecundity of *D. simulans* females from four PE-related genotypes over the course of 29 days at 23 °C. We found no difference among rates of fecundity decrease consistent with HD. (*B*) Decline of fecundity of *D. simulans* females from four PE-related genotypes over the course of 29 days at 29 °C. ♀M/P♂ F1 females have a lower intercept (i.e., initial fecundity) than the other three PE-related genotypes and also show a slightly more pronounced decrease in fertility as they age.

A third potential phenotype in HD is that PE carrying females show a rapid decrease in fertility as they age. Specifically, we tested whether the presence of PEs was predictive of reproductive output throughout the lifespan of females from crosses between P and M individuals. We tested this possibility by counting the number of ovarioles of F1 females from the four possible genotypes at six different ages for 29 days (fig. 5). We found no differences in rate of decay of fecundity at 23 °C (LRT: *χ*^2^ = 4.529, df = 3, *P* = 0.210; fig. 5*A*). At 29 °C, the intercept of the regression was lowest for ♀M/P♂ females which showed a lower fecundity at age 4 days, consistent with the observations at a single age described above (fig. 5*B*). We also observed a minor difference in the rate of decrease of ovariole number as females aged among genotypes (genotype × age effect: LRT; *χ*^2^ = 10.285, df = 3, *P* = 0.0163). ♀P/P♂, ♀M/M♂, and ♀P/M♂ females show the same rate of decrease of fecundity but ♀M/P♂ females show a slightly more pronounced decay in fecundity (estimate = −6.191 × 10^−3^, SE = 2.759 × 10^−3^, *Z* = −2.244, *P* = 0.025; fig. 5*B*). These results suggest that PEs in *D. simulans* might induce weak early reproductive senescence in ♀M/P♂ F1 females but only at high temperatures.

### The Number of PEs in the Paternal Genome Is Correlated with the Magnitude of HD in *D. simulans* and *D. melanogaster*

The experiments described above confirm that HD in *D. simulans* is not restricted to atrophied ovaries but also can manifest as a reduced number of ovarioles in nondysgenic ovaries. These are the same phenotypes that have been previously reported for *D. melanogaster* (Engels and Preston 1980; Khurana et al. 2011) and reviewed in Engels (1983) and Kelleher (2016). We also found that PEs can induce weak female reproductive senescence. Using two of these phenotypes (ovary number and ovariole number per ovary) and the estimation of the number of PEs per genome in *D. simulans* and *D. melanogaster*, we tested whether the number of PE copies in the genome was correlated with the strength of HD. We crossed two *D. simulans* lines (Riaba—a P line—and NC105—an M line—) to 37 *D. simulans* lines. We followed a similar approach for *D. melanogaster*; we crossed two *D. melanogaster* lines (Canton-S, an M line, and DGRP385, a P line) to 21 isofemale lines. We describe the results for each of these two phenotypes as follows.

#### Ovary Number

**Fig. 6.**
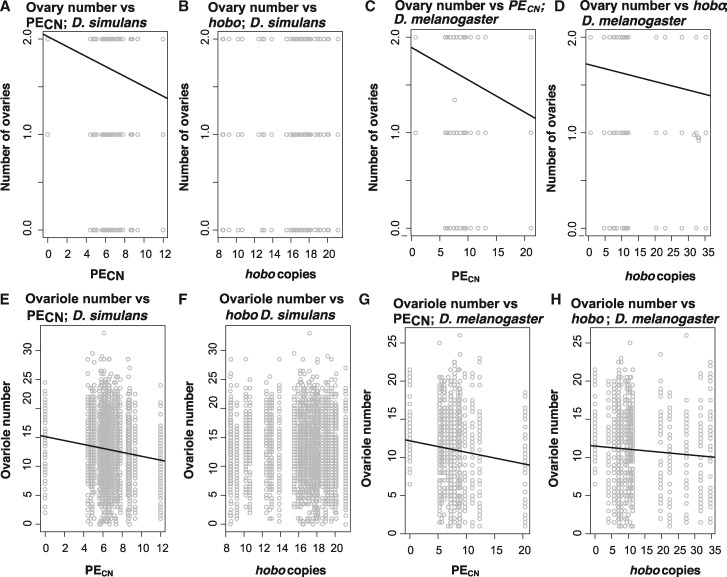
—The severity of HD increases with PE_CN_ in both *D. simulans* and *D. melanogaster* at 29 °C. (*A*–*D*) Relationships for ovary number and (*E*–*H*) relationships for ovariole number. (*A*, *B*, *E*, and *F*) Relationships in *D. simulans* and (*C*, *D*, *G*, and *H*) relationships in *D. melanogaster*. Only significant relationships have a trend line. Tables 1 and 2 show the coefficients and significance for each of these relationships.

First, we tested if PE copy number affects the number of ovaries in F1 females (scored as a multinomial outcome) in each of the two species. In the case of *D. simulans* raised at 23 °C, the effect of the number of PEs or *hobo* elements on the number of ovaries is undetectable regardless of the genotype of the female and of the method used to infer copy number (Illumina: table 1; Nanopore: supplementary table S5, Supplementary Material online). We found a similar trend in *D. melanogaster*; the number of PEs has no effect on the number of ovaries per female at 23 °C, regardless of the genotype of the female (table 1 and supplementary table S5, Supplementary Material online). At 29 °C, the effect of PE copy number is significant in both *D. melanogaster* and *D. simulans*, but when the mother is of the M type (table 1 and fig. 6*A*–*D*)*.* The effect of the number of *hobo* elements and the interaction between PE and *hobo* elements were significant in *D. melanogaster* but not in *D. simulans* (table 1). These results indicate that, as suggested previously for *D. melanogaster* (Srivastav and Kelleher 2017), the number of PE copies in the paternal genome does indeed affect the magnitude of HD but the relative importance of PE_CN_ on HD seems to differ between species.

**Table 1:** Effect of PE and *hobo* Copy Number in the Paternal Genomes on the Number of Ovaries per Female in *Drosophila simulans* and *Drosophila melanogaster* at Two Different Temperatures (23 and 29 °C)

Species	Mother Genotype	*T* (°C)	PE_CN_			*hobo*_CN_			PE_CN_ × *hobo*_CN_		
			**LRT (** *χ* **^2^)**	df	*P*	**LRT (** *χ* **^2^)**	df	*P*	**LRT (** *χ* **^2^)**	df	*P*
*D. simulans*	M	23	0.020	1	0.889	0.747	1	0.388	1.380	1	0.240
*D. simulans*	P	23	0.082	2	0.960	0.884	2	0.643	0.106	2	0.949
*D. simulans*	M	29	8.885	2	0.012	0.348	2	0.841	0.897	2	0.639
*D. simulans*	P	29	0.00	1	1	359.05	1	1	0.00	1	1
*D. melanogaster*	M	23	1.16 × 10^−3^	1	0.973	1.34 × 10^−5^	1	0.997	5.39 × 10^−3^	1	0.942
*D. melanogaster*	P	23	6.6 × 10^−4^	1	0.980	1.58 × 10^−4^	1	0.990	4.663 × 10^−3^	1	0.946
*D. melanogaster*	M	29	38.997	2	3.403 × 10^−9^	24.654	2	4.431 × 10^−6^	18.302	2	1.061 × 10^−4^
*D. melanogaster*	P	29	4.454	2	0.108	0.0226	2	0.989	0.140	2	0.933

Note.—We show LRT (*χ*^2^) and *P* values for each of the linear models (i.e., Poisson regressions) for each of the two species at two temperatures (a total of eight linear models). Odds ratios are shown in supplementary table S3, Supplementary Material online.

#### Ovariole Number

Next, we assessed whether PE and *hobo* numbers in the paternal genome also affected the number of ovarioles in F1 females from crosses between M and P individuals. For both species, the interaction between PE_CN_ and the mother genotype (i.e., whether the mother was of the P or M type) was not significant at 23 °C but was significant at 29 °C (table 2). The interaction between *hobo* copy number and the mother genotype had no effect *in D. simulans* or *D. melanogaster* at 23 °C but had an effect in *D. melanogaster* at 29 °C. Similarly, we found a strong interaction between the mother genotype, PE_CN_, and *hobo* copy number in *D. melanogaster* at 29 °C. Figure 6*E*–*H* shows the relationship between the number of ovarioles, PE_CN_ and *hobo* copy number in *D. simulans* (fig. 6*E* and *F*) and *D. melanogaster* (fig. 6*G* and *H*). These results indicate that, just as described with ovary number, mean ovariole number is affected by the number of PEs in the paternal genome in both *D. simulans* and *D. melanogaster*. Models that were systematically reduced showed similar results (supplementary table S6, Supplementary Material online). Results were also similar when we used only lines for which we counted the number of copies using only Nanopore sequencing (supplementary table S7, Supplementary Material online).

**Table 2:** Effect of PE and *hobo* Copy Number in the Paternal Genomes on Mean Ovariole Number per Ovary in *Drosophila simulans* and *Drosophila melanogaster*

	*D. simulans*						*D. melanogaster*					
	23			29			23			29		
	Coeff	LRT (*χ*^2^)	*P*	Coeff	LRT (*χ*^2^)	*P*	Coeff	LRT (*χ*^2^)	*P*	Coeff	LRT (*χ*^2^)	*P*
Genotype_mother_	0.146	1.093	0.296	−5.60 × 10^−2^	0.129	0.720	3.70 × 10^−2^	0.285	0.594	−0.184	6.243	0.013
PE_CN_	−9.16 × 10^−3^	0.479	0.489	−3.58 × 10^−2^	5.153	0.023	1.03 × 10^−2^	2.424	0.120	−6.94 × 10^−2^	79.165	<1 × 10^−10^
*hobo*_CN_	−2.56 × 10^−3^	0.134	0.714	1.06× 10^−2^	1.667	0.197	3.97 × 10^−3^	1.302	0.254	−2.87 × 10^−2^	41.919	<1 × 10^−10^
Genotype_mother_ × PE_CN_	−1.31 × 10^−2^	0.491	0.484	4.42 × 10^−2^	4.333	0.037	−4.10 × 10^−3^	0.192	0.662	7.61 × 10^−2^	53.857	<1 × 10^−10^
Genotype_mother_ × *hobo*_CN_	−1.14 × 10^−2^	1.325	0.250	−5.59 × 10^−3^	0.254	0.614	−0.55 × 10^−3^	0.270	0.603	3.29 × 10^−2^	33.553	<1 × 10^−10^
PE_CN_ × *hobo*_CN_	4.78 × 10^−4^	0.253	0.615	−1.12 × 10^−3^	0.973	0.324	−5.77 × 10^−4^	2.256	0.133	2.77 × 10^−3^	32.611	<1 × 10^−10^
Genotype_mother_ × PE_CN_ × *hobo*_CN_	1.0264 × 10^−3^	0.582	0.446	1.937 × 10^−4^	0.016	0.899	2.78 × 10^−4^	0.262	0.609	−3.10 × 10^−3^	24.554	<1 × 10^−10^

Note.—The table summarizes four linear models (two species × two temperatures). Coeff, coefficient; LRT, likelihood ratio test. We show LRT (*χ*^2^) and *P* values for each of the linear models (i.e., Poisson regressions) for each of the two species at two temperatures. For all effects, df was equal to 1.

### The Interaction between PE Copy Number in the Paternal Genome and Temperature Affects the Strength of HD

Our results indicate that HD is stronger at 29 °C than at 23 °C in both species. These results are in line with previous results that demonstrated that HD is stronger as temperature increases (Bingham et al. 1982; Hill et al. 2016). We expanded our studies on the effect of temperature on HD by studying the effect of PE copy number in causing HD in a larger range of temperature. We used a subset of lines (ten lines) and scored the number of ovaries and ovarioles per females in F1s produced from crosses to M and P females.

First, we studied the interaction of temperature and PE copy number in ovary number. In crosses involving *D. simulans* M females, the log odds ratio of having two functional ovaries versus having none decreased by 1.382 × 10^−2^ (*Z*-value = −4.274, *P* = 1.922 × 10^−5^). We did not calculate coefficients for crosses involving *D. simulans* P females, as all females resulting from this cross had two ovaries.

We observed a similar pattern in *D. melanogaster*. In crosses involving *D. melanogaster* M females, the log odds of having two functional ovaries versus having none decreased by 5.41 × 10^−3^ as PE_CN_ and temperature increase (*Z*-value = −2.742, *P* = 6.102 × 10^−3^). Puzzlingly, in crosses involving *D. melanogaster* P females, the log odds of having two functional ovaries versus having none increased slightly by 6.231 × 10^−3^ as PE_CN_ and temperature increase (*Z*-value = 1.983, *P* = 0.047).

Not surprisingly, we found that the interaction between parental genotypes (i.e., mother PE status and PE_CN_) and temperature affected the number of ovaries in F1 females from *D. melanogaster* crosses (LRT; *χ*^2^ = 127.4, df = 4, *P* < 1 × 10^−10^) and *D. simulans* (LRT; *χ*^2^ = 56.355, df = 4, *P* = 1.69 × 10^−11^). We observed no major signs of dysgenesis at temperatures lower than 25 °C and HD only manifested at temperatures higher than 25 °C, regardless of PE_CN_.

**Fig. 7.**
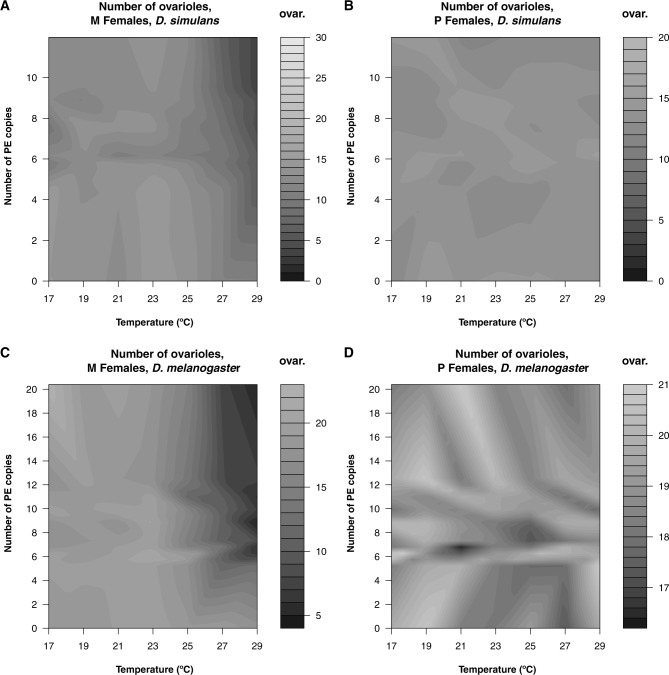
—The number of PEs and temperature interact to cause hybrid dysgenesis in both *D. simulans* and *D. melanogaster*. The heatmaps represent the number of ovarioles in F1 progeny from M females crossed to P males depending on paternal PE copy number and temperature. For each of the two species, we used ten different paternal lines which range in their PE copy number and raise the progeny of the crosses with an M and a P line at seven different temperatures. (*A*) Crosses involving *D. simulans* NC105 (M) females. (*B*) Crosses involving *D. simulans* MD199 (P) females. (*C*) Crosses involving *D. melanogaster* Canton-S (M) females. (*D*) Crosses involving *D. melanogaster* DGRP385 (P) females.

We found a similar trend in the mean number of ovarioles per ovary per female. The mean number of ovarioles for the two species and each type of cross is shown in figure 7. In the case of *D. simulans*, increasing PE_CN_ and temperature led to a decrease in the mean number of ovarioles in M females (coefficient = 1.431 × 10^−3^, standard error = 8.345 × 10^−5^, *Z*-value = −17.145, *P* < 1 × 10^−10^) and more modestly in P females (coefficient = −1.951 × 10^−4^, standard error = 7.716 × 10^−5^, *Z*-value = −2.529, *P* = 0.011). In the case of *D. melanogaster*, increasing PE_CN_ and temperature led to a decrease in the mean number of ovarioles in M females (coefficient = −1.020 × 10^−3^, standard error = 4.438 × 10^−5^, *Z*-value = −22.987, *P* < 1 × 10^−10^), but not in P females (coefficient = 3.358 × 10^−5^, standard error = 3.987 × 10^−5^, *Z*-value = −0.842, *P* = 0.40). Both of these interactions were significant (*D. simulans*: LRT; *χ*^2^ = 412.84, df = 2, *P* < 1 × 10^−10^; *D. melanogaster*: LRT; *χ*^2^ = 788.44, df = 2, *P* < 1 × 10^−10^). The *R*^2^ is significantly smaller for the *D. simulans* model (8.34%) than the *R*^2^ for the *D. melanogaster* model (9.84%; Wilcoxon test based on permutations: *Z* = 26.464, *P* < 1 × 10^−10^) suggesting that the interaction between the genotype of the mother, PE_CN_ in the paternal genome, and temperature is stronger in *D. melanogaster*.

## Discussion

PEs are arguably the best studied TE in animals; their phenotypic effects in *Drosophila* provide an ideal system to understand the organismal fitness effects during their spread within genomes. The most recently reported invasion of PEs occurred in *D. simulans*, where they lead to atrophied ovaries in ♀M/P♂ females (Hill et al. 2016), mirroring effects seen in *D. melanogaster* (Kidwell et al. 1977). In this report, we corroborate that HD in *D. simulans* is similar to the phenomenon of HD in *D. melanogaster* in that the phenomenon involves multiple phenotypes (as in Hill et al. [2016]). In both species, crosses between M females and P males produce dysgenic females with similar phenotypes, namely, reduced number of functional ovaries, and reduced number of mean ovarioles per ovary (Kidwell et al. 1977; Hill et al. 2016).

Our results are largely consistent with previous observations that PE_CN_ and HD are correlated (Engels et al. 1987, Hill et al. 2016, Srivastav SP, Kelleher ES. 2017). However, other studies have found that the proportion of dysgenic progeny is not correlated with PE_CN_ in ♀M/P♂ females (Srivastav et al. 2019). Discrepancy between studies might be analogous to the differences we find between species, with different genetic backgrounds from the same species differing in propensity to HD (as reported in Kelleher et al. 2018). Additionally, other TEs not studied here might also be involved in inducing HD, such as the *Har-P* PE variant in *D. melanogaster* which causes strong HD regardless of the PE_CN_ (Srivastav et al. 2019). Understanding the relationship between copy number and HD across genetic backgrounds will require a full exploration of the genomic regulators of PE transposition that cause HD.

Among the similarities between species, we find that PE_CN_ has a positive relationship with the severity of HD in both species (figs. 6 and 7). The molecular structure of PEs might explain the positive relationship between PE_CN_ and the severity of HD. All PEs have a canonical structure that includes 31-bp terminal inverted repeats (TIR) and internal inverted repeats of 11 bp located about 100 bp from the ends that interact with the THAP domain of the transposase (Beall and Rio 1997; Lee et al. 1998; Majumdar and Rio 2015). When a PE invades a new species, all individuals are of the M type. As the PE increases in frequency, each PE insertion generates a new TIR site, which in turn provides a substrate for the P-encoded endonuclease to cut. As the number of TIRs increases, the likelihood of DNA damage and dysgenesis might also increase. This progression is only stopped if some PE copies are incorporated into a piRNA cluster, which silences TEs and suppresses HD (Obbard et al. 2009; Lu and Clark 2010; Rozhkov et al. 2013; Kofler 2019), or the population goes extinct (Charlesworth B and Charlesworth D 1983; Arkhipova and Meselson 2005). Whether the number of TIR copies in the genome explains the positive relationship between the severity of HD and PE_CN_ remains untested.

Notably, we also find differences between species. First, we find that PEs might have weak role on F1 female reproductive senescence in *D. simulans*. This effect has not been reported in *D. melanogaster* but given the subtle nature of the defect, it might have gone previously unnoticed. Second, the interaction between PE copy number, temperature, and maternal genotype seem to be more pronounced in *D. melanogaster* than in *D. simulans*. The genome of *D. melanogaster* has a higher TE content than *D. simulans* (Vieira and Biémont 2004; Lerat et al. 2011; Kofler, Nolte, et al. 2015; Adrion et al. 2019) but in the particular case of PEs, the two species have similar copy numbers. This is intriguing as the invasion of *D. simulans* is more recent than that of *D. melanogaster* (Kofler, Hill, et al. 2015). The TEs in the two species genomes seem to differ in their transposition rates (Kofler, Nolte, et al. 2015), which might explain why PEs are more deleterious in *D. melanogaster* than in *D. simulans*. The function of at least one Piwi protein has diverged these two species (i.e., *aubergine*; Kelleher et al. 2012) by natural selection (Simkin et al. 2013), which might explain differences in the phenomenon of HD between species. The only previous study comparing the magnitude of HD in two species of *Drosophila*, studied the spread of PEs in mixed populations containing both M and P individuals of *D. simulans* and *D. melanogaster*. (P lines of *D. simulans* were generated by genetic transformation.) PEs showed a higher rate of transposition in *D. melanogaster* than *D. simulans* which was attributed to interspecific differences in unknown genetic factors (Kimura and Kidwell 1994). Our results are consistent with this observation as we found that the joint effect of PE_CN_ and temperature on HD is stronger in *D. melanogaster* than in *D. simulans*.

More research is warranted to understand differences in the phenomenon of HD between species. In *D. melanogaster*, transpositional insertions into piRNA clusters in the genome of the mother suppress TE movement (reviewed by Kelleher [2016]), and in the case of PEs suppress gonadal atrophy. A single TE insertion in a piRNA cluster may be sufficient for repressing the activity of a TE (Ronsseray et al. 1989; Josse et al. 2007; Zanni et al. 2013). There is evidence of pervasive positive selection in genes in the Piwi pathway (Simkin et al. 2013). In *D. melanogaster*, piRNA clusters have evolved rapidly in the recent past through positive selection (Zhang and Kelleher 2019). PE-induced gonadal atrophy is also affected by multiple QTLs in the genome, of which *bruno* has the strongest effect size (Kelleher et al. 2018). *bruno* affect the strength of HD by modulating germline stem cell loss in the presence of PE activity (Kelleher et al. 2018). *Har-P* PEs can cause strong HD regarldess of the PE_CN_ (Srivastav et al. 2019). No study has yet addressed whether this also occurs in *D. simulans* or even more distantly related species, but there is variability in the phenomenon of HD in *D. simulans*.

Although it is clear that PEs have increased their frequency in both *D. simulans* and *D. melanogaster*, little is known about the progression of the copy number per species and its associated effects. Experimental evolution experiments in *D. simulans* have suggested that the outcome of invasions might be predictable and contingent on temperature. At high temperatures (mean: ∼23 °C, range: [18–28 °C]), PEs spread rapidly from 1.79 copies per genome to an average of 31.7 copies per genome after just 20 generations. At this point, the infection plateaued in terms of copy number (Kofler et al. 2018). Some of these PEs were internally deleted, which shows how quickly PEs can degenerate after an invasion and calls into question how fast PEs degenerate in different species. Although there was also a monotonic increase in the total number of PE copies per population at cool temperatures (mean: ∼15 °C, [10–20 °C]) over 40 generations, populations did not reach the same level of PE copy number as the populations reared in hot conditions. These results indicate that temperature has an effect not only on the strength of HD but also on the rate at which PEs invade a population. Experimental evolution experiments, as the one done in *D. simulans*, have the potential to reveal whether PEs are equally likely to invade different species, whether the rate of degeneration and suppression is similar across species, and the influence of environmental conditions on TE invasions.

Our results are consistent with previous observations that have shown that HD is a complex phenomenon that depends on the mother genotype, the copy number of PEs, and temperature. We also find that the strength of HD depends on the species identity and the interactions between these multiple factors. Other epistatic interactions, and gene × environment interactions, are likely to play a role on the phenomenon but remain to be studied. Studying the genomic features that might affect the strength of HD, besides the presence and absence of PEs, is an opportunity to understand the similarities and differences of how PEs coevolve with their hosts’ genomes (Kelleher et al. 2018).

## Supplementary Material

evaa084_Supplementary_Data

## Notes

Data deposition: All raw read data are available via the NCBI Sequence Read Archive (BioProject accession: PRJNA622255).  Raw data and analytical code have been deposited at Dryad under the accesion: https://doi.org/10.5061/dryad.w3r2280mr.
