# Supplementary material for: Paternally Inherited P-Element Copy Number Affects the Magnitude of Hybrid Dysgenesis in Drosophila simulans and D. melanogaster
Source: Genome Biol Evol. 2020 Apr 27;12(6):808–26. doi: 10.1093/gbe/evaa084 (PMC7313671; doi:10.1093/gbe/evaa084)

**Paternally inherited P-element copy number affects the magnitude of hybrid dysgenesis in *Drosophila simulans* and *D. melanogaster***

Antonio Serrato-Capuchina, Jeremy Wang, Eric Earley, David Peede, Kristin Isbell, and Daniel R. Matute

**SUPPLEMENTARY MATERIAL**

**TABLES S1-S7**

**TABLE S1.** **Details of isofemale lines used in this study.**

**TABLE S2. Lines sequenced with Oxford nanopore Technologies.**

**TABLE S3. Multinomial regression coefficients for ovary number in *D. simulans*.**

**TABLE S4. ♀M × M♂ F1 hybrids have fewer ovaries that other female genotypes.**

**TABLE S5. Effect of PE_CN_ and *hobo* copy number in the paternal genomes on the number of ovaries per female in *D. simulans* and *D. melanogaster* at two different temperatures (23ºC and 29ºC)*.***

**TABLE S6. Effect of PE_CN_ and *hobo* copy number in the paternal genomes on mean ovariole number per ovary in *D. simulans* and *D. melanogaster.***

**TABLE S7. Effect of PE_CN_ and *hobo* copy number in the paternal genomes on mean ovariole number per ovary in *D. simulans* and *D. melanogaster.***

**FIGURES S1-S7**

**FIGURE S1. Identification of full and partial TE insertions by long read sequencing.**

**FIGURE S2. Incomplete PE elements are common in *D. simulans* genomes.**

**FIGURE S3. Incomplete PE elements are common in *D. melanogaster* genomes.**

**FIGURE S4.** **Incomplete PEs are common in *D. wilinstoni*.**

**FIGURE S5. Incomplete *hobo* elements are common in *D. simulans* genomes.**

**FIGURE S6. Incomplete *hobo* elements are common in *D. melanogaster* genomes.**

**FIGURE S7. PE insertion site frequency spectrum in *D. simulans.***

**SUPPLEMENTARY TABLES**

**TABLE S1.** **Details of isofemale lines used in this study.** Lines in bold were used in the experiments to determine the effect of copy number in the paternal genome in the magnitude of hybrid dysgenesis. Sequencing coverage refers only to coverage for short reads.

| **Species** | **Name** | **Collection date** | **Collection place** | **Sequencing coverage** | **SRA number** |
| --- | --- | --- | --- | --- | --- |
| *D. simulans* | 18BOMA03 | 2018 | Kenya | 48.152 | SRR11456805 |
| *D. simulans* | 18boma1 | 2018 | Kenya | 50.596 | SRR11456804 |
| *D. simulans* | 18CHAALES15 | 2018 | Kenya | 29.185 | SRR11456793 |
| *D. simulans* | 18KARI01 | 2018 | Kenya | 30.054 | SRR11456782 |
| *D. simulans* | 18KARI03 | 2018 | Kenya | 54.707 | SRR11456771 |
| *D. simulans* | 18KARI04 | 2018 | Kenya | 39.872 | SRR11456760 |
| *D. simulans* | 18KARI05 | 2018 | Kenya | 48.533 | SRR11456753 |
| *D. simulans* | 18KARI06 | 2018 | Kenya | 37.372 | SRR11456752 |
| *D. simulans* | 18KARI09 | 2018 | Kenya | 28.039 | SRR11456751 |
| *D. simulans* | 18KARI10 | 2018 | Kenya | 32.352 | SRR11456750 |
| *D. simulans* | 18KARI26 | 2018 | Kenya | 35.579 | SRR11456803 |
| *D. simulans* | 18KARI37 | 2018 | Kenya | 39.000 | SRR11456802 |
| *D. simulans* | 18KARITANA20 | 2018 | Kenya | 41.990 | SRR11456801 |
| *D. simulans* | 18MPALA02 | 2018 | Kenya | 26.860 | SRR11456800 |
| *D. simulans* | 18MPALA02B | 2018 | Kenya | 45.641 | SRR11456799 |
| *D. simulans* | 18MPALA02C | 2018 | Kenya | 53.852 | SRR11456798 |
| *D. simulans* | 18MPALA03 | 2018 | Kenya | 24.683 | SRR11456797 |
| *D. simulans* | 18MPALA05 | 2018 | Kenya | 43.950 | SRR11456796 |
| *D. simulans* | 18MPALA08 | 2018 | Kenya | 55.148 | SRR11456795 |
| *D. simulans* | 18MPALA09 | 2018 | Kenya | 44.621 | SRR11456794 |
| *D. simulans* | 18MPALA11 | 2018 | Kenya | 39.935 | SRR11456792 |
| *D. simulans* | 18MPALA12 | 2018 | Kenya | 31.995 | SRR11456791 |
| *D. simulans* | 18MPALA13 | 2018 | Kenya | 44.523 | SRR11456790 |
| *D. simulans* | 18MU04 | 2018 | Senegal | 53.835 | SRR11456789 |
| *D. simulans* | 18MU05 | 2018 | Senegal | 33.599 | SRR11456788 |
| *D. simulans* | 18NANY04 | 2018 | Senegal | 32.965 | SRR11456787 |
| *D. simulans* | 18NANY10 | 2018 | Senegal | 47.431 | SRR11456786 |
| *D. simulans* | 18NANY15 | 2018 | Senegal | 42.639 | SRR11456785 |
| *D. simulans* | 18NANY16 | 2018 | Senegal | 44.693 | SRR11456784 |
| *D. simulans* | BiokoH9 | 2013 | Equatorial Guinea | 64.097 | SRR11456783 |
| *D. simulans* | BiokoLB1 | 2013 | Equatorial Guinea | 82.236 | SRR11456781 |
| *D. simulans* | cascade1 | 2013 | Equatorial Guinea | 66.026 | SRR11456780 |
| *D. simulans* | H1 | 2013 | Equatorial Guinea | 68.847 | SRR11456779 |
| *D. simulans* | KARI25 | 2018 | Kenya | 32.081 | SRR11456778 |
| *D. simulans* | Md199 | unknown | Madagascar | 94.272 | SRR11456777 |
| *D. simulans* | Riaba | 2013 | Equatorial Guinea | 63.979 | SRR11456776 |
| *D. simulans* | Riaba9 | 2013 | Equatorial Guinea | 65.868 | SRR11456775 |
| *D. melanogaster* | BCM-DGRP208 | | USA (DGRP) | 117.681 | SRS003442 |
| *D. melanogaster* | melMW56 | ~1990 | Malawi | 37.875 | SRR11456774 |
| *D. melanogaster* | BCM-DGRP301 | | USA (DGRP) | 117.681 | SRX005978 |
| *D. melanogaster* | BCM-DGRP303 | | USA (DGRP) | 258.596 | SRX005986 |
| *D. melanogaster* | BCM-DGRP304 | | USA (DGRP) | 227.960 | SRX005988 |
| *D. melanogaster* | BCM-DGRP315 | | USA (DGRP) | 100.934 | SRX006143 |
| *D. melanogaster* | BCM-DGRP324 | | USA (DGRP) | 203.732 | SRX006145 |
| *D. melanogaster* | BCM-DGRP385 | | USA (DGRP) | 22.727 | SRX159098 |
| *D. melanogaster* | melLA66 | ~1990 | Botswana | 56.211 | SRR11456773 |
| *D. melanogaster* | melMW11 | ~1990 | Malawi | 39.982 | SRR11456772 |
| *D. melanogaster* | melMW28 | ~1990 | Malawi | 40.715 | SRR11456770 |
| *D. melanogaster* | melOK91 | ~1990 | Botswana | 39.710 | SRR11456769 |
| *D. melanogaster* | melZH16 | ~1990 | Zimbabwe | 54.439 | SRR11456768 |
| *D. melanogaster* | melZH27 | ~1990 | Zimbabwe | 41.244 | SRR11456767 |
| *D. melanogaster* | melZH32 | ~1990 | Zimbabwe | 44.611 | SRR11456766 |
| *D. melanogaster* | melZS29 | ~1990 | Zimbabwe | 40.921 | SRR11456765 |
| *D. melanogaster* | melZS53 | ~1990 | Zimbabwe | 38.324 | SRR11456764 |
| *D. melanogaster* | melZS56 | ~1990 | Zimbabwe | 41.557 | SRR11456763 |
| *D. melanogaster* | melZS6 | ~1990 | Zimbabwe | 32.952 | SRR11456762 |
| *D. melanogaster* | melZS8 | ~1990 | Zimbabwe | 70.401 | SRR11456761 |
| *D. melanogaster* | NMB15_35 | 2015 | Zambia | 26.571 | SRR11456759 |

**TABLE S2. Lines sequenced with Oxford nanopore Technologies.**

| **Line** | **Species** | **SRA** |
| --- | --- | --- |
| Cascade1 | *D. simulans* | SRR11456756 |
| H9 | *D. simulans* | SRR11456758 |
| H1 | *D. simulans* | SRR11456755 |
| Riaba | *D. simulans* | SRR11456754 |
| LB1 | *D. simulans* | SRR11456757 |
| NC105 | *D. simulans* | SRR11480907 |

**TABLE S3. Multinomial regression coefficients for ovary number in *D. simulans*.** The response had three possible outcomes (0,1, or 2 functional ovaries).

| **Multinomial linear regression: ovary number 23ºC/ P mother** | | | | |
| --- | --- | --- | --- | --- |
| **Odds-ratios** | **Intercept** | ***PE_CN_*** | ***hobo_CN_*** | ***PE_CN_* × *hobo_CN_*** |
| 0 vs. 1 | 10.870 | 1.419 | -0.704 | -0.055 |
| 0 vs. 2 | 25.772 | 0.651 | -1.261 | 0.008 |
| **Multinomial linear regression: ovary number 29ºC/ P mother**  Pos mother | | | | |
| **Odds-ratios** | **Intercept** | ***PE_CN_*** | ***hobo_CN_*** | ***PE_CN_* × *hobo_CN_*** |
| 0 vs. 1 | NA | NA | NA | NA |
| 0 vs. 2 | NA | NA | NA | NA |
| **Multinomial linear regression: ovary number 23ºC/ M mother** | | | | |
| **Odds-ratios** | **Intercept** | ***PE_CN_*** | ***hobo_CN_*** | ***PE_CN_* × *hobo_CN_*** |
| 0 vs. 1 | 0.482 | 39.005 | 0.2016142 | -1.863 |
| 0 vs. 2 | 4.208 | 39.205 | 0.6449798 | -1.926 |
| **Multinomial linear regression: ovary number 29ºC/ M mother** | | | | |
| **Odds-ratios** | **Intercept** | ***PE_CN_*** | ***hobo_CN_*** | ***PE_CN_* × *hobo_CN_*** |
| 0 vs. 1 | 1.330 | -0.144 | 0.026 | -0.008 |
| 0 vs. 2 | 3.844 | -0.415 | -0.019 | 0.002 |

**TABLE S4. ♀M × M♂F1 hybrids have fewer ovaries that other female genotypes.** We did pairwise comparisons between genotypes using a approximative two-sample Fisher-Pitman permutation Tests.

|  | **Mean** | **SD** | **♀M × M♂** | **♀M × P♂** | **♀P × M♂** | **♀P × P♂** |
| --- | --- | --- | --- | --- | --- | --- |
| **♀M × M♂** | 2.00 | 0.00 | - | 8.42 | NA | NA |
| **♀M × P♂** | 1.50 | 0.74 | < 1 × 10^-10^ | - | -8.42 | -8.42 |
| **♀P × M♂** | 2.00 | 0.00 | NA | NA | - | NA |
| **♀P × P♂** | 2.00 | 0.00 | NA | NA | NA | - |

**TABLE S5. Effect of PE_CN_ and *hobo* copy number in the paternal genomes on the number of ovaries per female in *D. simulans* and *D. melanogaster* at two different temperatures (23ºC and 29ºC)*.*** The analysis and results are similar to the ones shown in Table 1 but here we used the inferred number of *PE* and *hobo* copies with Nanopore sequenced instead of short reads.

| **Species** | **Mother** | **T (ºC)** | ***PE_CN_*** | | | ***hobo_CN_*** | | | ***PE_CN_* ×*hobo_CN_*** | | |
| --- | --- | --- | --- | --- | --- | --- | --- | --- | --- | --- | --- |
|  |  |  | ***LRT (X^2^)*** | **df** | **P** | ***LRT (X^2^)*** | **df** | **P** | ***LRT (X^2^)*** | **df** | **P** |
| *D. simulans* | M | 23 | 3.057 × 10^-5^ | 1 | 0.996 | 6.301 × 10^-4^ | 1 | 0.980 | 0.000 | 1 | 1 |
| *D. simulans* | P | 23 | NA | NA | NA | NA | NA | NA | NA | NA | NA |
| *D. simulans* | M | 29 | 20.316 | 2 | 3.877 × 10^-5^ | 0.653 | 2 | 0.723 | 9.776 | 2 | 7.538 × 10^-3^ |
| *D. simulans* | P | 29 | NA | NA | NA | NA | NA | NA | NA | NA | NA |

**TABLE S6. Effect of PE_CN_ and *hobo* copy number in the paternal genomes on mean ovariole number per ovary in *D. simulans* and *D. melanogaster.*** This table corresponds to Table 2 after systematic model reduction. Only significant effects are shown. R: reduced.

|  |  |  | | *D. simulans* | | |  |  |  | *D. melanogaster* | | |
| --- | --- | --- | --- | --- | --- | --- | --- | --- | --- | --- | --- | --- |
|  |  | 23 | |  | 29 | |  | 23 |  |  | 29 | |
|  | Coeff | LRT (*X*^2^) | P | Coeff | LRT (*X*^2^) | P | Coeff | LRT (*X*^2^) | P | Coeff | LRT (*X*^2^) | P |
| Genotype_mother_ | R | R | R | 0.086 | 13.961 | 1.867× 10^-4^ | R | R | R | -0.184 | 6.243 | 0.013 |
| *PE_CN_* | R | R | R | 0.045 | 154.901 | < 1× 10^-10^ | R | R | R | -6.94 × 10^-2^ | 79.165 | < 1 × 10^-10^ |
| *hobo_CN_* | R | R | R | R | R | R | R | R | R | -2.87× 10^-2^ | 41.919 | < 1 × 10^-10^ |
| Genotype_mother_ × *PE_CN_* | R | R | R | 0.049 | 69.420 | < 1× 10^-10^ | R | R | R | 7.61 × 10^-2^ | 53.857 | < 1 × 10^-10^ |
| Genotype_mother_×*hobo_CN_* | R | R | R | R | R | R | R | R | R | 3.29× 10^-2^ | 33.553 | < 1 × 10^-10^ |
| *PE_CN_* × *hobo_CN_* | R | R | R | R | R | R | R | R | R | 2.77× 10^-3^ | 32.611 | < 1 × 10^-10^ |
| Genotype_mother_× *PE_CN_* × *hobo_CN_* | R | R | R | R | R | R | R | R | R | -3.10× 10^-3^ | 24.554 | < 1 × 10^-10^ |

**TABLE S7. Effect of *PE* and *hobo* copy number in the paternal genomes on mean ovariole number per ovary in *D. simulans* and *D. melanogaster.*** Coeff : Coefficient. LRT: Likelihood ratio test. The analysis and results are similar to the ones shown in Table 2 but here we used the inferred number of *PE* and *hobo* copies with Nanopore sequenced instead of short reads.

|  | ***D. simulans*** | | | | | |
| --- | --- | --- | --- | --- | --- | --- |
|  | **23** | | | **29** | | |
|  | Coeff | LRT (*X*^2^) | P | Coeff | LRT (*X*^2^) | P |
| Genotype_mother_ | 1.475 | 1 | 0.225 | 1.514 | 1 | 0.219 |
| *PE_CN_* | 4.716 | 1 | 0.030 | 17.134 | 1 | 3.484× 10^-5^ |
| *hobo_CN_* | 0.021 | 1 | 0.886 | 0.135 | 1 | 0.713 |
| Genotype_mother_ × *PE_CN_* | 7.233 | 1 | 0.007 | 21.196 | 1 | 4.146 × 10^-6^ |
| Genotype_mother_ ×*hobo_CN_* | 0.064 | 1 | 0.800 | 0.347 | 1 | 0.556 |
| *PE_CN_*× *hobo_CN_* | 4.488 | 1 | 0.034 | 10.103 | 1 | 1.481× 10^-3^ |
| Genotype_mother_ × *PE_CN_* × *hobo_CN_* | 6.051 | 1 | 0.014 | 15.154 | 1 | 9.907× 10^-5^ |

**SUPPLEMENTARY FIGURES**

**FIGURE S1. Identification of full and partial TE insertions by long read sequencing.** The reference genome (with no PEs, red), canonical PE sequence (green), and aligned reads (blue, reverse-complement: light blue). The left panel shows a case of a full-length insertion in which the long read covers the whole PE sequence. The right panel shows an inverted partial copy retaining only exons 1 and 4 (right). In this example, each insertion event is supported by four long reads.


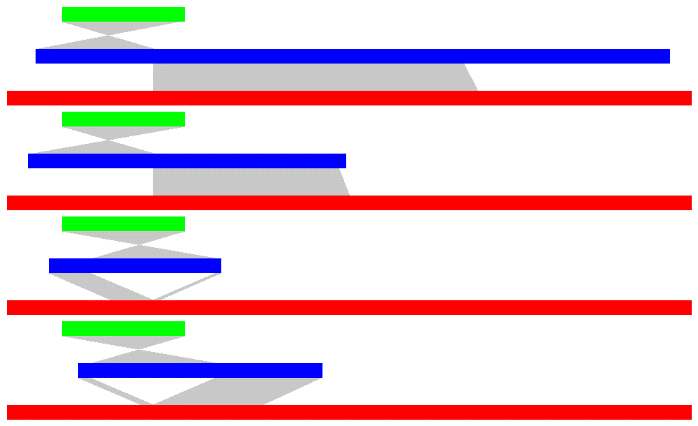

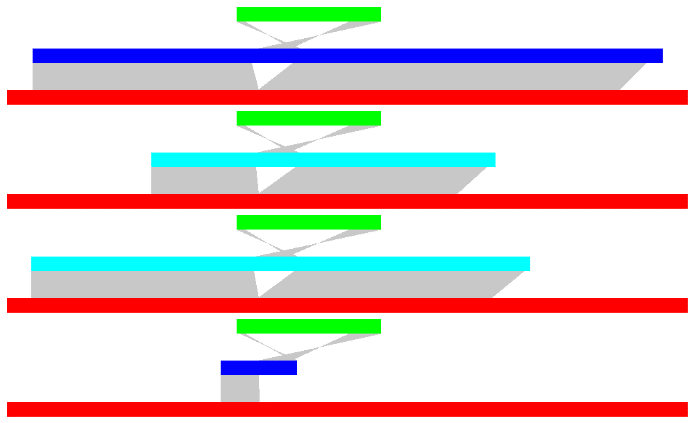


**FIGURE S2. Incomplete PE elements are common in *D. simulans* genomes.** The *y*-axis shows the mean coverage per site along the two elements. Each panel shows the coverage profile of an isofemale line. Panel F appears also appears as Figure 3A and was included for reference.

**FIGURE S3. Incomplete PE elements are common in *D. melanogaster* genomes.** The *y*-axis shows the mean coverage per site along the two elements. Each panel shows the coverage profile of an isofemale line. Panel A appears also appears as Figure 3B and was included for reference.

**FIGURE S4.** **Incomplete PEs are common in *D. wilinstoni*.** The *y*-axis shows the mean coverage per site for the canonical *D. melanogaster* P-element sequence.


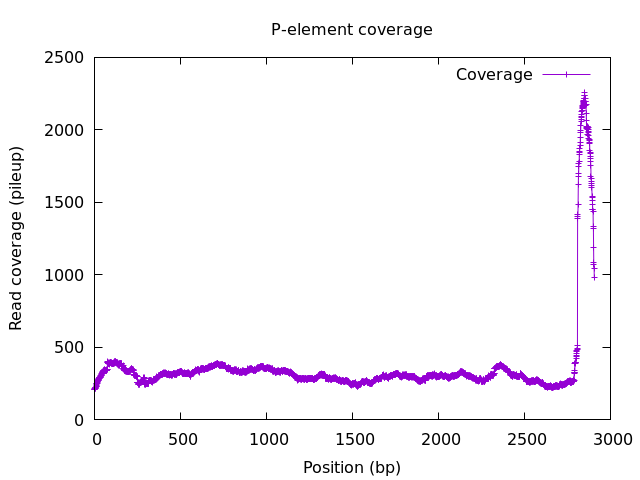


**FIGURE S5. Incomplete *hobo* elements are common in *D. simulans* genomes.** The *y*-axis shows the mean coverage per site along the two elements. Each panel shows the coverage profile of an isofemale line. Panel F appears also appears as Figure 3C and was included for reference.

**FIGURE S6. Incomplete *hobo* elements are common in *D. melanogaster* genomes.** The *y*-axis shows the mean coverage per site along the two elements. Each panel shows the coverage profile of an isofemale line. Panel A appears also appears as Figure 3D and was included for reference,

**FIGURE S7. PE insertion site frequency spectrum in *D. simulans.*** Most insertions appear as singletons. We did not plot the site frequency spectrum for *D. melanogaster* because of the lack of Nanopore data for our samples.


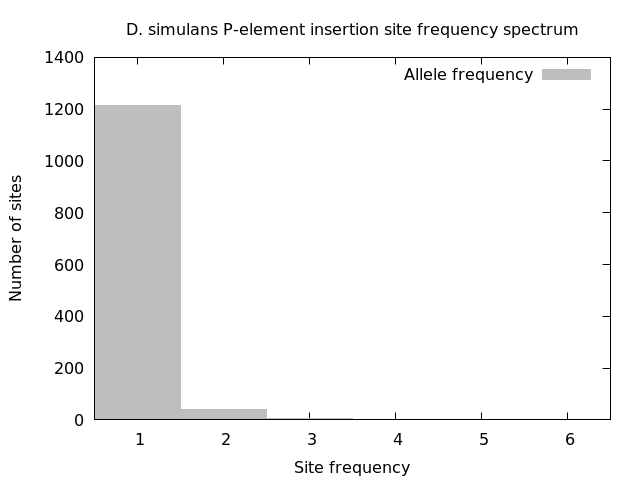

Supplement: evaa084_Supplementary_Data [file evaa084_supplementary_data.docx]
